# The timing of frugivore‐mediated seed dispersal effectiveness

**DOI:** 10.1111/mec.14850

**Published:** 2018-09-17

**Authors:** Juan P. González‐Varo, Juan M. Arroyo, Pedro Jordano

**Affiliations:** ^1^ Integrative Ecology Group Estación Biológica de Doñana, EBD‐CSIC Sevilla Spain; ^2^ Terrestrial Ecology Group Instituto Mediterráneo de Estudios Avanzados UIB‐CSIC Esporles Spain

**Keywords:** birds, DNA barcoding, frugivory, seasonal variation, seedling recruitment, species interactions

## Abstract

The seed dispersal effectiveness framework allows assessing mutualistic services from frugivorous animals in terms of quantity and quality. Quantity accounts for the number of seeds dispersed and quality for the probability of recruitment of dispersed seeds. Research on this topic has largely focused on the spatial patterns of seed deposition because seed fates often vary between microhabitats due to differences in biotic and abiotic factors. However, the temporal dimension has remained completely overlooked despite these factors—and even local disperser assemblages—can change dramatically during long fruiting periods. Here, we test timing effects on seed dispersal effectiveness, using as study case a keystone shrub species dispersed by frugivorous birds and with a fruiting period of 9 months. We evaluated quantity and quality in different microhabitats of a Mediterranean forest and different periods of the fruiting phenophase. We identified the bird species responsible for seed deposition through DNA barcoding and evaluated the probability of seedling recruitment through a series of field experiments on sequential demographic processes. We found that timing matters: The disperser assemblage was temporally structured, seed viability decreased markedly during the plant's fruiting phenophase, and germination was lower for viable seeds dispersed in the fruiting peak. We show how small contributions to seed deposition by transient migratory species can result in a relevant effectiveness if they disperse seeds in a high‐quality period for seedling recruitment. This study expands our understanding of seed dispersal effectiveness, highlighting the importance of timing and infrequent interactions for population and community dynamics.

## 1 INTRODUCTION

Mutualistic interactions constitute an essential element of biodiversity that provides key ecological functions, from mycorrhizal‐mediated mineral nutrition to animal‐mediated pollination and seed dispersal (Jordano, 2016; Schupp, Jordano, & Gómez, 2017). A major challenge in understanding the role of mutualistic interactions in community dynamics lies in assessing not only the *immediate* outcome*,* but also the *delayed* effect that interacting species have on their partners (Schupp et al., 2017). The immediate outcome is the successful occurrence of interactions and can be largely assessed as a quantity component (number of events; for example, number of seeds dispersed). The delayed outcome is the “per capita” effect a species has on the demography of its interacting partner and can be assessed as a quality component (e.g., probability of recruitment of a dispersed seed). This framework allows the total effect of interactions to be estimated for both sides of the mutualism as the product of quantity and quality (quantity × quality), which results in a measure of effectiveness (Schupp et al., 2017).

Seed dispersal mediated by frugivorous animals is a central process in the dynamics and regeneration of many vegetation types (Herrera, 2002; Jordano, 2013; Wang & Smith, 2002). The effectiveness framework has provided a conceptual and analytical tool for the study of seed dispersal mutualisms from the plant's perspective for more than two decades (Schupp, 1993; Schupp, Jordano, & Gómez, 2010). Research on seed dispersal effectiveness has largely focused both on gut passage effects and on the spatial patterns of seed deposition generated by different disperser species, with consequences for recruitment (Jordano & Schupp, 2000; Schupp, 1993; Schupp et al., 2010). Gut passage effects on germination can vary among groups of seed dispersers (Nogales et al., 2017; Traveset, 1998), while the fates of seeds and seedlings often differ between microhabitats and habitat types due to spatial variation in biotic and abiotic factors, such as seed predator activity, irradiance, soil humidity or intra‐ and interspecific competition (Gómez‐Aparicio, 2008; González‐Varo, Nora, & Aparicio, 2012; Rey & Alcántara, 2014; Schupp, 1995). The latter explains why space has been a major factor when considering the quality of seed dispersal services provided by different animal partners (Calviño‐Cancela & Martín‐Herrero, 2009; Escribano‐Ávila et al., 2014; Rother et al., 2016; Schupp et al., 2010). The quantity component has been assessed either by combining information on microhabitat use by different disperser species with measures of seed deposition across microhabitats (Donoso, García, Rodríguez‐Pérez, & Martínez, 2016; Jordano & Schupp, 2000) or through visual identification of dispersers from droppings with seeds (only feasible with taxonomically distant dispersers; e.g., Calviño‐Cancela & Martín‐Herrero, 2009; McConkey, Brockelman, & Saralamba, 2014). The quality component has been assessed either by field experiments of seed survival, germination and seedling establishment (Escribano‐Ávila et al., 2014) or by monitoring these demographic processes in naturally dispersed seeds and seedlings (Donoso et al., 2016). These studies have shown how effective dispersers can compensate a modest quantity component with seed deposition in high‐quality sites for recruitment (Calviño‐Cancela & Martín‐Herrero, 2009; Escribano‐Ávila et al., 2014; McConkey et al., 2014).

**Figure 1:**
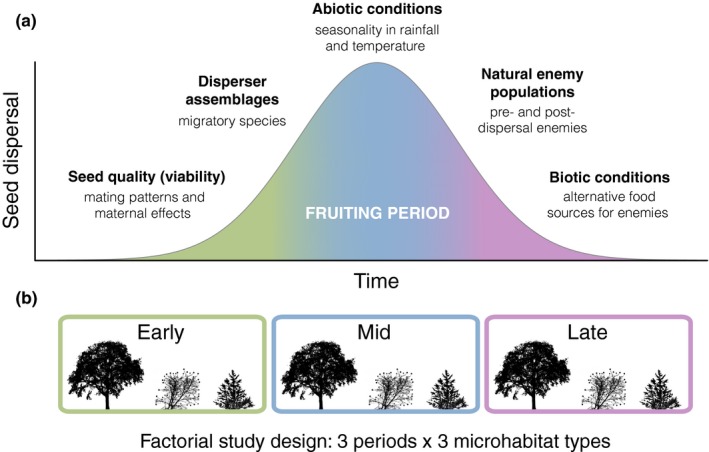
(a) Biotic and abiotic factors important for seed dispersal effectiveness that can vary substantially during the whole fruiting period of plant species; their position along the *x*‐axis does not indicate their timing. The *y*‐axis indicates the number of dispersed seeds; and the bell‐shaped curve is merely hypothetical and illustrative (temporal distributions could be asymmetric or multimodal). (b) Study design to address temporal variation in seed dispersal effectiveness through an assessment of quantity and quality components in different periods of the fruiting phenology and across the same target microhabitats [Colour figure can be viewed at http://wileyonlinelibrary.com]

Surprisingly, the temporal dimension, in terms of between‐ and within‐season variability, has remained completely overlooked in the study of seed dispersal effectiveness. The fruiting period of many plants dispersed by animals can last for many months (Hamann, 2004; Snow & Snow, 1988) and even for most of the year (Herrera, 1984), a common phenomenon in tropical regions (Griz & Machado, 2001; Peres, 1994). During such long periods, the biotic and abiotic factors affecting seed dispersal and seedling recruitment can change dramatically (Figure 1a). First, the local disperser assemblage can be temporally structured during the fruiting period because many migratory animals are frugivores, mostly birds and bats (e.g., Herrera, 1984; Stiles, 1980; Thomas, 1983). This involves that the contribution of a migrant species to the dispersal of a given plant species can be confined to a particular, narrow temporal window. Moreover, the populations seed predators (or parasites) can fluctuate within and between seasons (Ostfeld & Keesing, 2000), as well as their predation pressure on a given seed species due to changes in the abundance of alternative food resources (García, Martínez, & Obeso, 2007; Price & Joyner, 1997). In addition, climatic seasonality can determine more or less suitable periods for seedling emergence and survival, particularly in highly seasonal ecosystems (Garwood, 1983; Gómez‐Aparicio, 2008; Washitani & Masuda, 1990). Lastly, even the intrinsic quality of seeds might vary between early‐ and late‐ripening fruits owing to resource limitation (Vaughton & Ramsey, 1998) or to the activity of different pollinator species during the plant's flowering phenology (Ivey, Martinez, & Wyatt, 2003; Valverde, Gómez, & Perfectti, 2016). All these sources of temporal variability (Figure 1a) suggest that, in many plant species and across biomes, the timing of seed dispersal could be as crucial modulating seed dispersal effectiveness as the seed deposition sites. There could be also interactive “space–time” effects shifting the relative quality of microhabitats throughout the fruiting period. Tackling this issue empirically is challenging and requires answering the questions *who, where* and *when* dispersed the seeds, and *what* happened to them next.

Here, we test timing effects on seed dispersal effectiveness. We used as study case a Mediterranean shrub dispersed by frugivorous birds whose fruiting period can last for up to 9 months. We approached the spatiotemporal variation in effectiveness using a factorial study design (“period × microhabitat”) that allowed us to evaluate the quantity and quality components in different microhabitats and different periods of the plant's fruiting phenophase (Figure 1b). We identified the bird species responsible for seed deposition (i.e., quantity) through DNA barcoding applied on dispersed seeds—animal DNA can be successfully extracted from the surface of defecated or regurgitated seeds collected in the field (González‐Varo, Arroyo, & Jordano, 2014; González‐Varo, Carvalho, Arroyo, & Jordano, 2017). We evaluated the probability of recruitment (i.e., quality) through a series of field experiments on sequential demographic processes (seed viability, seed predation, germination and seedling survival). Then, for three groups of birds differing in their migratory behaviour, we assessed how seed dispersal effectiveness varied in space and time, and the importance of accounting for timing when estimating the overall effectiveness throughout the whole fruiting phenophase.

## 2 MATERIALS AND METHODS

### 2.1 The plant–frugivore system

The study plant species was the lentisc (*Pistacia lentiscus*, Anacardiaceae), an evergreen dioecious shrub with hemispherical shape (Supporting Information Figure S1), that constitutes a dominant component of woodlands and forests in warm, lowland areas across the Mediterranean Basin (http://www.worldwildlife.org/biomes; Yi, Wen, Golan‐Goldhirsh, & Parfitt, 2008). We chose this species because its fruiting phenology can last up to 9 months, from late summer to early spring (Jordano, 1989), a long period during which many biotic and abiotic factors important for seed dispersal effectiveness vary considerably (Figure 1a, Supporting Information Figure S1). Its single‐seeded fruits are spherical drupes of ~5 mm in diameter with a lipid‐rich pulp (Herrera, 1987). Fruits are red prior to ripening and black when ripe (Supporting Information Figure S1). Its lentil‐shaped seeds are 3–5 mm in diameter and 2–3 mm in width. A significant proportion of the fruits contain unviable seeds due to abortion, parthenocarpy or parasitism by *Megastigmus pistaciae*, a chalcidoid wasp (see Supporting Information Figure S1; Jordano, 1989; Verdú & García‐Fayos, 1998). Fruits can remain red throughout the fruiting season because colour is also associated with seed viability and most red fruits contain unviable seeds (Jordano, 1989). Lentisc fruits are consumed—and its seeds dispersed—by a diverse guild of small frugivorous birds, mainly belonging to families Sylviidae, Turdidae and Muscicapidae, which includes resident birds, sub‐Saharan migrants and European wintering migrants (Herrera, 1984; Jordano, 1988, 1989). The lentisc has an ephemeral seed bank because its seeds lack dormancy and germinate within the year (García‐Fayos & Verdú, 1998).

### 2.2 Study site

We conducted our study in Garrapilos, a Mediterranean lowland forest of ca. 120 ha located in southern Spain (Cádiz province; 36°39.6′N, 5°56.9′W). Its vegetation consists of large holm (*Quercus ilex* subsp. *ballota*) and cork (*Q. suber*) oaks (10–12 m height), and an understorey dominated by treelets and shrubs (2–4 m height), among which wild olive trees (*Olea europaea* var. *sylvestris*), kermes oaks (*Q. coccifera*, Fagaceae), lentiscs, evergreen buckthorns (*Rhamnus alaternus*, Rhamnaceae) and hawthorns (*Crataegus monogyna*, Rosaceae) are the dominant species (Supporting Information Figure S1). A lower layer of scrubs (<1 m height) is dominated by rockroses (*Cistus salviifolius*, Cistaceae). The mean lentisc cover was 30%, and the mean cover of the main vegetation elements was as follows: oak trees 31%, shrubs 49%, scrubs 15% and uncovered soil (both with and without oak canopy above) 36% (cover data from 20 × 30‐m line transects); only uncovered soil, shrubs and scrubs account for 100% because the tree cover can overlap with these elements. Our sampling area covered ca. 20 ha within this forest.

### 2.3 Sampling design

We studied different demographic processes associated with the quantity and quality components of seed dispersal effectiveness in three microhabitat types replicated in three periods of the fruiting phenology (early, mid and late), following a factorial study design (Figure 1b). We divided the 9‐month fruiting period (August–April) previously observed in the study site into three 3‐month periods classified as *early* (August–October), *mid* (November–January) and *late* (February–April) of the lentisc fruiting phenology. These same‐sized temporal frames allow the magnitude of seed dispersal to be compared between periods. We expected this period length (3 months) to properly capture local turnover in disperser species (see Section 2.5 and Supporting Information Table S1) and to include contrasting climatic conditions that are important for seedling recruitment (the mid‐period is generally colder and wetter than the early and late periods; Supporting Information Figure S2). We evaluated the quantity of seed deposition as the contribution of different disperser species to seed rain across “microhabitat–period” combinations (Table 1). For the quality sub components, we assessed variation in seed viability only between periods, whereas we assessed post dispersal processes (survival to seed predation, germination and seedling survival) across “microhabitat–period” combinations (Table 1). We focused on three microhabitat types (Figure 1b): (a) on uncovered soil under the canopy of oak trees (*trees*, hereafter); (b) under treelets/shrubs bearing fleshy fruits (*fruit‐bearing shrubs*, hereafter); and (c) under shrubs not bearing fleshy fruits (*non‐fb shrubs*, hereafter); these microhabitats accounted for 53% cover in the study site (8%, 17% and 28%, respectively). We chose these microhabitats because birds typically use trees and shrubs as perches, dropping most seeds beneath them (Izhaki, Walton, & Safriel, 1991; Jordano & Schupp, 2000; Rey & Alcántara, 2014); in fact, we have found that lentisc seed rain densities on open ground are negligible (González‐Varo et al., 2014) and very low beneath *Cistus* scrubs (J.P. González‐Varo unpublished data). Besides, germination and establishment of lentisc seedlings are favoured beneath trees and shrubs due to favourable microclimatic conditions produced under their canopy (Verdú & García‐Fayos, 1996). We differentiated between types of shrubs because bird‐generated seed rain is generally higher beneath fruit‐bearing plants (Herrera, Jordano, López‐Soria, & Amat, 1994; Montesinos, Verdú, & García‐Fayos, 2007) and also because post dispersal processes such as seed predation can be both conspecific and heterospecific density‐dependent (García et al., 2007; Kwit, Levey, & Greenberg, 2004). The observational and experimental procedures to assess different demographic process are specified below.

**Table 1:** Demographic processes assessed in this study belonging to the quantity or quality components of seed dispersal effectiveness (SDE). We assessed variation in these processes between disperser species (D), periods (P) within the fruiting phenology of the plant and microhabitats (M) of seed arrival and seedling recruitment

SDE component	Demographic process	Metric	Factors
Quantity	Seed deposition	Seed rain density (seeds/m^2^)	D*,* P*,* M
Quality	Seed viability	Proportion of viable seeds	P
Quality	Escape to seed predation	Proportion of seeds that survive	P*,* M
Quality	Germination	Proportion of seeds that germinate	P*,* M
Quality	Seedling survival^a^	Proportion of seedlings that survive	P*,* M

Note

a Seedling survival until early autumn after the first summer.

### 2.4 Bird‐mediated seed dispersal

We sampled lentisc seeds dispersed by birds in the study site during the whole 2014–2015 fruiting season, from summer 2014 to spring 2015 (August–April; 9 months in total). We used seed traps placed beneath the three target microhabitats (*trees*,* fruit‐bearing shrubs* and *non‐fb shrubs*) to quantify the magnitude of seed deposition. Seed traps consisted of plastic trays (40 cm × 55 cm, 8 cm height) with small holes (1 mm diameter) to allow the drainage of rainwater and covered with wire mesh (1 cm × 1 cm) to prevent post dispersal seed predation by vertebrates (Supporting Information Figure S1). We monitored a total of 37 seed traps placed beneath different oak trees (*n *=* *12), treelets/shrubs bearing fleshy fruits (*n *=* *13; 5 wild olive trees, 4 female lentiscs and 4 hawthorns) and shrubs not bearing fleshy fruits (*n *=* *12; 4 kermes oaks, 4 male lentiscs and 4 male evergreen buckthorns); distance between seed traps ranged from 5 to 530 m. We conducted sampling surveys fortnightly where we recorded the number of bird‐dispersed lentisc seeds per seed trap and sampled individual seeds or droppings for DNA barcoding analysis (see Section 2.5). We did so putting each sample with a minimum of handling into a 1.5‐ml sterile tube with the aid of the tube cap. Tubes were labelled and stored in a freezer at −20°C until DNA extraction (González‐Varo et al., 2014). In each sampling survey, we generally sampled either all or most seeds from those seed traps receiving few seeds (1–4), whereas we generally sampled a subsample from seed traps receiving many seeds (>5). Overall, we sampled 44% of all seeds found in the seed traps (457 out of 1,030 seeds).

### 2.5 Seed disperser identification through DNA barcoding

We used DNA barcoding to identify the bird species that dispersed the seeds sampled (*n* = 457 seeds in 443 samples; 13 samples contained 2–3 seeds in the same bird dropping). DNA of animal origin can be extracted from the surface of defecated or regurgitated seeds, allowing the identification of the frugivore species responsible of each dispersal event (González‐Varo et al., 2014, 2017). Briefly, disperser species identification was based on a 464‐bp mitochondrial DNA region (COI: cytochrome *c* oxidase subunit I). For DNA extraction, we used a GuSCN/silica protocol, incubating each seed directly in extraction buffer (added to the 1.5‐ml tube where the seed was sampled in the field) (see details in González‐Varo et al., 2014). For PCR amplification, we used the primers COI‐fsdF (5′–GCATGAGCCGGAATAGTRGG–3′) and COI‐fsdR (5′–TGTGAKAGGGCAGGTGGTTT–3′) following the PCR protocol described by González‐Varo et al. (2014). For a subset of sampled seeds (ca. 10%) that failed to amplify using COI‐fsd primer pair, we used an additional protocol using other primer sets to gain in amplification success for smaller DNA fragments. Details are provided in González‐Varo et al. (2017). Briefly, this protocol consisted of nested PCRs, using a new primer set designed for shorter sequences (COI‐fsd‐degR: 5′–GTTGTTTATTCGGGGGAATG–3′, to be combined with COI‐fsdF; COI‐fsd‐degF: 5′–GGAGCCCCAGACATAGCAT–3′, to be combined with COI‐fsdR) (González‐Varo et al., 2017) on the amplicon AWCintF2–AWCintR4 (avian DNA barcodes; Lijtmaer, Kerr, Stoeckle, & Tubaro, 2012) as template (following Alcaide et al., 2009).

We only sequenced one strand (forward primer) of the amplified COI fragments because in most cases the electrophoretic patterns were clear and resulting sequences (length: mean = 365 bp; median = 397 bp; range = 104–417 bp) allowed successful discrimination between species. Sequences (i.e., barcodes) were aligned and edited using sequencher 4.9, and then identified using the “barcode of life data” identification system (bold: http://www.boldsystems.org; Ratnasingham & Hebert, 2007). bold accepts sequences from the 5′ region of the COI gene and returns species‐level identification and assigns a percentage of similarity to matched sequences.

We classified the DNA‐identified bird species as *residents*,* sub‐Saharan migrants* and *European migrants* in order to analyse whether the quantity and quality components of seed dispersal effectiveness are dependent on birds’ migratory strategy. Sub‐Saharan migrants include species that either breed in the study area or use it as a stopover during their autumn migration to Africa. European migrants include species that overwinter in the study area. Classification was based on the online Encyclopaedia of the Birds of Spain (http://www.seo.org/listado-aves) and the species’ occurrences in the study site, which were assessed through monthly bird censuses (Supporting Information Table S1).

### 2.6 Pre dispersal loses: Seed viability

We evaluated differences between periods in the viability (%) of bird‐dispersed seeds sampled in the field through the “flotation/sink” method: Only seeds that sink have a well‐developed embryo inside (validated by Albaladejo, González‐Martínez, Heuertz, Vendramin, & Aparicio, 2009). We conducted this test when the extraction buffer for DNA barcoding analysis was added to the tube containing the seed. We expected temporal differences in seed viability because (a) the ratio between red and black fruits varies through the fruiting season; (b) fruits can remain red in colour because colour is also associated with seed viability and most red fruits contain unviable seeds; and (c) although birds have a strong preference for black fruits, they also consume red ones (Herrera, 1984; Jordano, 1989).

We also evaluated the viability of seeds (%) inside black fruits in order to obtain viable seeds for the sowing experiments (see Section 2.6). We collected black fruits from nine mother plants in the early period (October), 10 in the mid‐period (December) and 11 in the late period (February). We collected fruits from the same mother plants, whenever possible, to minimize maternal effects. However, some plants did not have black fruits in the late period, so we had to sample fruits of neighbour plants (shared mother plants between periods: early–mid: 9; early–late: 4; mid–late: 5). We collected a total of 2,288 black fruits (555–925 per period), for which we tested the viability of depulped seeds through the “flotation/sink” method. We opened a subset of seeds that floated (*n *=* *147) and corroborated that they were either empty (69%; due to abortion or parthenocarpy) or contained a wasp larva (31%) (Supporting Information Figure S1).

### 2.7 Post dispersal fates: Seed predation, germination and seedling establishment

We assessed three post dispersal processes (seed predation, germination and seedling establishment) through experiments conducted in the nine “microhabitat–period” combinations of our study design (Figure 1, Table 1). The experiments were set up on October 17, 2014, December 23, 2014, and February 27, 2015, for the periods *early*,* mid* and *late*, respectively (see Supporting Information Figure S2). The experiments were conducted in an area of ~0.5 m^2^ under the canopy of individual trees or shrubs, which represented the microhabitat replicates.

We assessed post dispersal seed predation by rodents in each study period by placing experimental units (seed depots, hereafter) across the three target microhabitats. Seed depots consisted of 10 lentisc seeds glued firmly to one side of a 10 cm × 10 cm of green plastic mesh (1‐mm pore size), which was nailed to the ground (see Rey et al., 2002). In each period, we placed 7–10 seed depots per microhabitat (i.e., 23–26 per period; *n*_total_ = 75 depots with 750 seeds), which were monitored only after 2 weeks due to very high predation rates (see Section 3). We considered a seed to have been preyed upon if it disappeared from the square or if it was still on the square but was gnawed and empty (Supporting Information Figure S1).

Seedling emergence and survival were assessed through a sowing experiment, which is known to be a robust tool for disentangling processes affecting seedling establishment (González‐Varo et al., 2012). In each period, we sowed seeds in the three target microhabitats to assess seed germination and seedling survival. All lentisc seeds used in this experiment were viable seeds collected from 9 to 11 mother plants per period (see details in Section 2.6). Seeds were sown in 7–10 replicated stations per microhabitat (i.e., 22–26 per period; *n*_total_ = 71 stations with 639 seeds sowed). In each station, we removed any naturally dispersed seeds and then sowed nine seeds uniformly distributed in a matrix of 3 rows × 3 columns, separated by 5 cm, at a depth of 0.5–1 cm. We added litter to match the natural conditions as close as possible and protected the sowing stations with a wire mesh cage (with a grid of 15 × 15 cm area and 15 cm height; 1 cm × 1 cm) to prevent predation by rodents (e.g., González‐Varo et al., 2012). Additionally, we added thin wire mesh (1‐mm pore size) on the top of the cage to prevent the deposition of lentisc seeds into the sowing stations (Supporting Information Figure S1). Seed germination and seedling survival were monitored fortnightly during the first ~4 months after sowing and monthly thereafter until October 28, 2015. We chose early autumn, after the first summer faced by the seedlings, as the end of the experiment because seedling survival to the summer drought is a crucial process affecting recruitment dynamics across Mediterranean plant species (Gómez‐Aparicio, 2008).

### 2.8 Data analyses

All analyses were performed using the r computing environment v. 3.3.3 (R Core Team, 2015). We used different types of models (according to the data structure and the nature of the response variable) to test for significant effects of period, microhabitat and their interaction (P × M) on the demographic processes studied. The interaction term allowed us to test whether the effects of the period were consistent across microhabitats. We used a linear model (LM) to test for spatiotemporal differences in seed rain density (seeds/m^2^), which was ln (*x *+* *1) transformed to meet the normality and homoscedasticity assumptions. We used binomial distributions and logit link functions to analyse seed viability, seed predation, seed germination and seedling survival, all of which were Bernoulli‐distributed response variables (1 =  success, 0 = failure). For seed viability, we used a generalized linear model (GLM) to test for the effect of period. For seed predation, seed germination and seedling survival, we used generalized linear mixed models (GLMM) where period and microhabitat were included as fixed effects and the identity of the experimental stations (i.e., seed depots or sowing stations) was included as a random effect (Bolker et al., 2009). GLMMs were fitted using the package lme4 (v. 1.1–12) (Bates, Maechler, Bolker, & Walker, 2014), and the significance of fixed effects (*p*‐values of Wald χ^2^ tests) was computed using the “Anova” function of the package car (v. 2.1–6) (Fox & Weisberg, 2011).

For the analyses of seed dispersal effectiveness, we grouped the bird species contributing to seed rain (DNA‐identified) by their migratory strategy (i.e., residents, sub‐Saharan migrants and European migrants; see Section 2.5). We did so because: these groups are expected to contribute to seed dispersal in different periods of the fruiting phenology (Figure 1a); the sub‐Saharan migrants included many species but with small contributions to the lentisc seed rain (see Section 3); all bird species within and across groups were relatively similar in terms of body size (i.e., small passerines of 12–70 g); and for the sake of simplicity because 3 bird groups × 3 periods × 3 microhabitats already account for 27 potential combinations of effectiveness. We calculated the quantity and quality components of seed dispersal effectiveness for different bird species groups (*i*) contributing to seed dispersal across the study periods (*j*) and microhabitats (*k*). We calculated the quantity component as follows:

$$QT_{\mathrm{ijk}}=d_{\mathrm{jk}}\times f_{\mathrm{ijk}}$$

where *d*_*jk*_ is the magnitude of seed deposition (i.e., seed rain density) in period *j* and microhabitat *k*, and *f*_*ijk*_ is the relative contribution (frequency) of bird species group *i* to period *j* and microhabitat *k*. In other words, the quantity of seed deposition contributed by each bird species group in each “microhabitat–period” combination. We calculated the quality component as follows:

$$QL_{\mathrm{jk}}=v_{j}\times p_{\mathrm{jk}}\times g_{\mathrm{jk}}\times s_{\mathrm{jk}}$$

where *v*_*j*_ is the probability of viability among bird‐dispersed seeds in period *j*, whereas *p*_*jk*_, *g*_*jk*_ and *s*_*jk*_ are, respectively, the probabilities of escaping to post dispersal seed predation, germinating and surviving as seedling for seeds dispersed in period *j* and microhabitat *k*. In other words, QL is the cumulative probability of recruitment of dispersed seeds in each “microhabitat–period” combination (see Table 1). We obtained zero probabilities in most *p*_*jk*_ and in two *s*_*jk*_, which likely reflected sample size limitations to accurately measure these demographic processes, rather than they were fully collapsed. For operational purposes, we replaced these zeros with low values in order to avoid zeros in the computed ql_*jk*_ (e.g., González‐Varo et al., 2012; Rey & Alcántara, 2014). First, we assigned a constant probability of *p*_*jk*_ = 0.01 (1%) because predation rates showed no variability and were almost total across periods and microhabitats. Second, we conservatively replaced the two zero values obtained for *s*_*jk*_ with the minimum non zero value we obtained for the probability of seedling survival (*s* = 0.09) across “microhabitat–period” combinations. We calculated the seed dispersal effectiveness for each bird species group contributing to seed dispersal across periods and microhabitats as follows:

$${\text{SDE}}_{\mathrm{ijk}}=QT_{\mathrm{ijk}}\times QL_{\mathrm{jk}}$$

We also calculated the overall effectiveness across periods (SDE_*ik*_ = qt_*ik*_ × ql_*k*_), where qt_*ik*_ is the sum of qt_*ijk*_ across periods for each “bird species group–microhabitat” combination, and ql_*k*_ is the weighted mean of ql_*jk*_ across periods (weighted by qt_*ijk*_); the reason for weighting is that the cumulative probabilities of recruitment represented by ql_*jk*_ were associated with different fractions of seeds arriving to each microhabitat. We used the package effect.lndscp (v. 0.2.8) (by P. Jordano; see Schupp et al., 2017) to represent “quantity × quality” effectiveness landscapes.

## 3 RESULTS

### 3.1 Seed rain density and frugivore contributions

**Figure 2:**
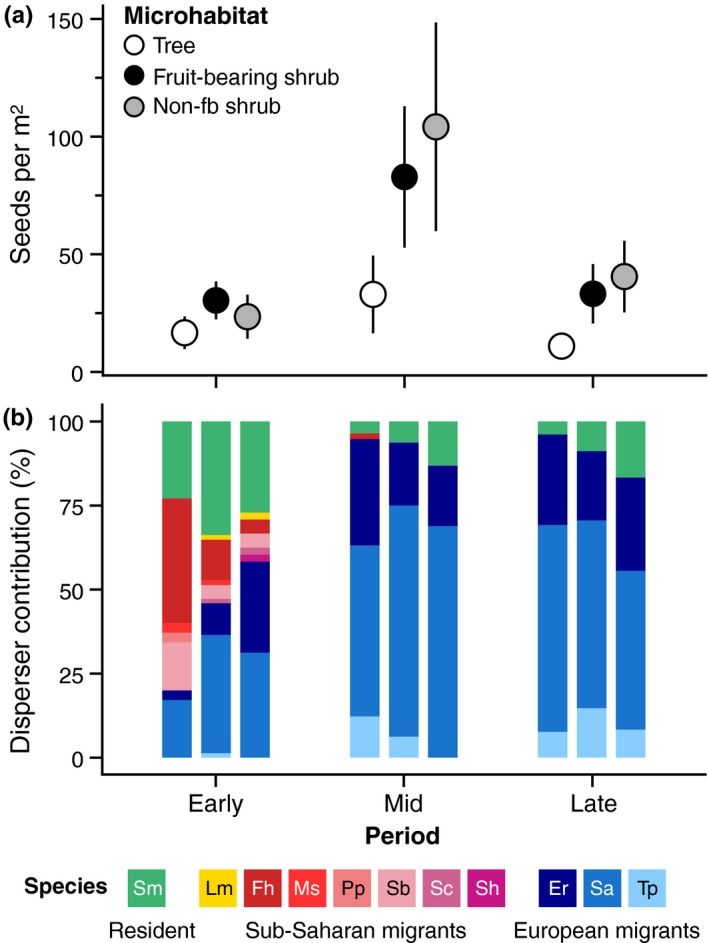
(a) Frugivore‐mediated seed rain density (mean ± 95% CI) of *Pistacia lentiscus* in three microhabitat types for each of the three study periods of the 2014–2015 fruiting season. (b) Relative contribution (%) to seed rain densities by the 11 bird species identified through DNA barcoding applied to defecated/regurgitated seeds (*n*_total_ = 435 seeds; mean = 48, range = 26–74 per “microhabitat–period” combination). Full bird species names: *Sylvia melanocephala* (Sm), *Luscinia megarhynchos* (Lm), *Ficedula hypoleuca* (Fh), *Muscicapa striata* (Ms), *Phoenicurus phoenicurus* (Pp), *Sylvia borin* (Sb), *Sylvia communis* (Sc), *Sylvia hortensis* (Sh), *Erithacus rubecula* (Er), *Sylvia atricapilla* (Sa) and *Turdus philomelos* (Tp). According to their migratory strategy, these species included resident birds (1 species), sub‐Saharan migrants (7 species) and European migrants (3 species) [Colour figure can be viewed at http://wileyonlinelibrary.com]

We found that seed rain density mediated by birds varied significantly between periods (Table 2). Not surprisingly, the greatest seed densities were found in the mid‐period, with values that were ~3 times higher than in the early and late periods, when densities were very similar (Figure 2a). Seed rain density also varied significantly between microhabitats (Table 2), with the highest values found beneath both types of shrubs (with and without fruits) and the lowest beneath trees (~2–3 times lower; Figure 2a). The non significant interaction term of the LM indicated that the differences observed between periods were consistent across microhabitats (Table 2).

**Table 2:** Significance of the fixed factors of our sampling design (“period,” “microhabitat type” and their interaction) on models analysing the demographic processes studied (*df*: degrees of freedom)

Response	Model	Tests	Period		Microhabitat type		P × M	
			*df* = 2	*p*	*df* = 2	*p*	*df* = 4	*p*
Seed rain	LM	*F*	18.5	**1.4** × **10** ^**−7**^	19.3	**8.0** × **10** ^**−8**^	1.7	0.146
Seed viability^a^	GLM	Wald χ^2^	43.5	**3.5** × **10** ^**−10**^	–	–	–	–
Seed predation^b^	GLMM	Wald χ^2^	0.0	1.000	<0.1	0.999	0.0	0.999
Germination	GLMM	Wald χ^2^	20.8	**3.0** × **10** ^**−5**^	2.2	0.327	4.9	0.300
Seedling survival	GLMM	Wald χ^2^	2.6	0.457	7.3	*0.063*	0.8	0.936

Note

*p*‐Values < 0.05 are shown in bold; a *p*‐value < 0.10 is shown in italics.

LM: linear model; GLM: generalized linear model (binomial); GLMM: generalized linear mixed models (binomial with seed depot or sowing station as random factor).

**Figure 3:**
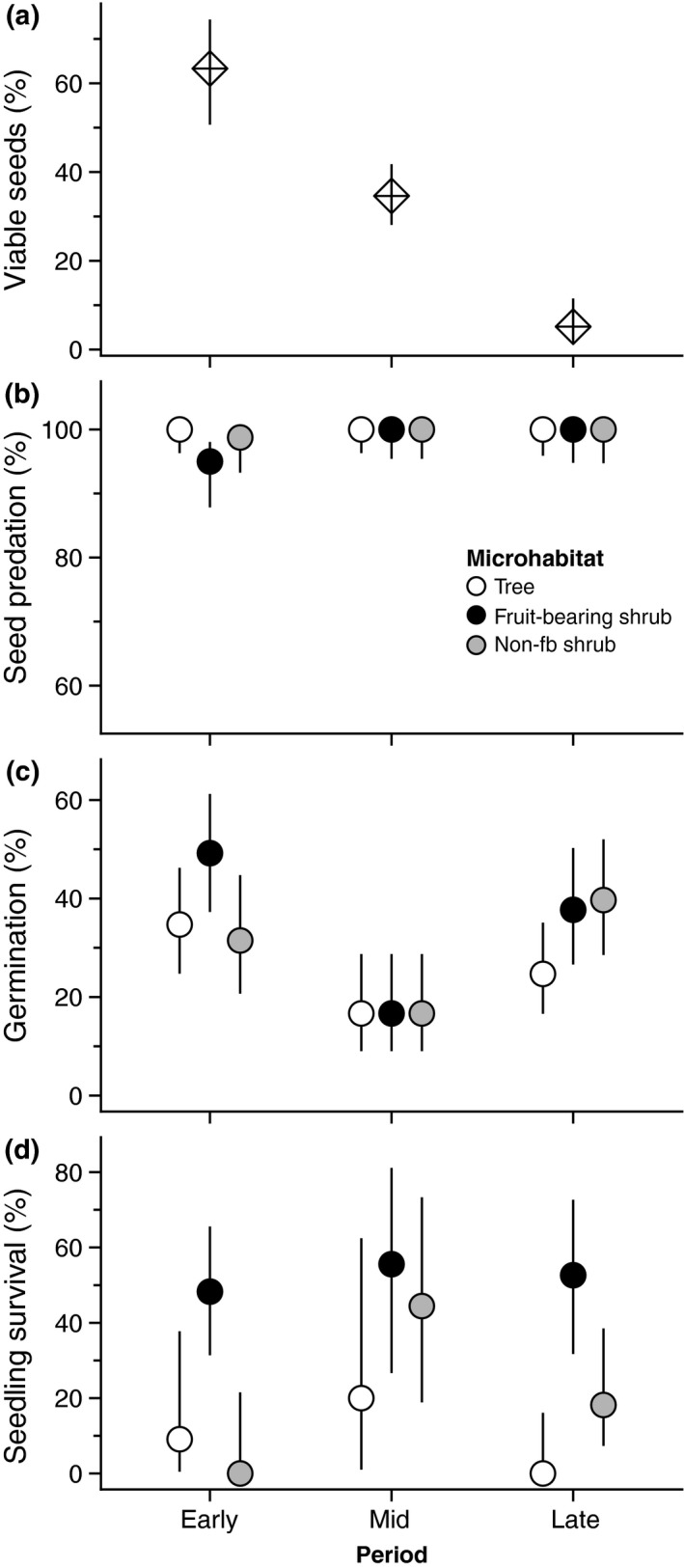
Variation across periods (a–d) and microhabitats (c,d) in demographic processes of *Pistacia lentiscus* belonging to the qualitative component of seed dispersal effectiveness. Symbols and vertical bars represent, respectively, observed percentages and 95% binomial confidence intervals. (a) Viability of dispersed seeds (*n*_total_ = 339 seeds). (b) Post dispersal seed predation by vertebrates (*n*_total_ = 749 seeds). (c) Germination of viable seeds (*n*_total_ = 556 seeds). (d) Seedling survival until the early autumn after the first summer (*n*_total_ = 138 seedlings)

^a^Differences in seed viability were only assessed between periods. ^b^Seed predation was 100% in 73 of the 75 seed depots placed in the field (see Figure 3b).

We successfully identified through DNA barcoding a total of 11 bird species from 422 samples (435 seeds) of the total 443 samples analysed (457 seeds); that is, the disperser was successfully identified in 95.3% of samples (PCR amplification failed in the remaining 4.7%). These 11 species included one resident bird, seven sub‐Saharan migrants and three European migrants (Figure 2b). Remarkably, DNA barcoding tools allowed us to identify seed dispersal by two sub‐Saharan migrants (*Sylvia communis* and *Sylvia hortensis*) that were not recorded in the bird censuses (see Supporting Information Table S1). The resident bird and the European migrants contributed to seed rain in all “microhabitat–period” combinations, whereas sub‐Saharan migrants only contributed in four combinations, and mostly in the early period (Figure 2b). Indeed, seed dispersal in the early period was mediated by a diverse avian assemblage (11 species), and seed rain contributions were evenly distributed between residents, and sub‐Saharan and European migrants (Figure 2b). In contrast, seed dispersal in periods mid and late was mediated by a simpler avian assemblage (4 species), and seed rain contributions were dominated by European migrants (83–96%; Figure 2b). The early period also showed the highest differences between microhabitats in seed rain contributions: Most seeds deposited beneath trees were dispersed by sub‐Saharan migrants (57%), whereas most deposited beneath non‐fruit‐bearing shrubs were dispersed by European migrants (58%).

### 3.2 Pre dispersal loses: Seed viability

We found a striking decrease in the viability of dispersed seeds through the fruiting phenology (Figure 3a). Mean viability dropped from 63% in the early period to 35% in the mid‐period and then to 5% in the late period (Figure 3a). Accordingly, period has highly significant effects in the GLM (Table 2). We found a parallel decrease in seed viability for ripe fruits collected from lentisc plants (see Supporting Information Figure S3), yet viability rates were higher (82%, 63% and 17% in periods early, mid and late, respectively).

### 3.3 Post dispersal fates: Seed predation, germination and seedling establishment

We found huge post dispersal seed predation rates, which were above 95% across all “microhabitat–period” combinations and complete (100%) in most combinations (Figure 3b); only 5 of the 750 seeds survived the experiment after 2 weeks. Some sowing stations were lost due to damage by wild boars (*Sus scrofa*): Nine stations (13%) were lost from the germination data and 18 stations (25%) from the survival data (see details in Supporting Information Table S2). We recorded a total of 168 emerged seedlings in the 62 sowing stations (556 seeds) comprising the germination data. Seed germination varied significantly between periods but not between microhabitats, and the non significant interaction term of the GLMM indicated that the differences observed between periods were also consistent across microhabitats (Table 2). Germination rates were higher in periods early and late than in the mid‐period (Figure 3c). Such higher germination rates were associated with germination speeds, as these were significantly faster in periods early and late (mean = 8.1 and 6.9 weeks after sowing, respectively) than in the mid‐period (mean = 11.3 weeks; see details in Supporting Information Figure S4). A total of 39 seedlings survived until the end of the experiment in the 53 sowing stations (138 seedlings) comprising the survival data. Seedling survival showed more erratic patterns, both across periods and microhabitats, although the highest survival was always found beneath fruit‐bearing shrubs (Figure 3d). Period and microhabitat had non significant effects on the GLMM (*p *>* *0.4), but the interaction term was marginally significant (*p *=* *0.063; Table 2).

### 3.4 Seed dispersal effectiveness

**Figure 4:**
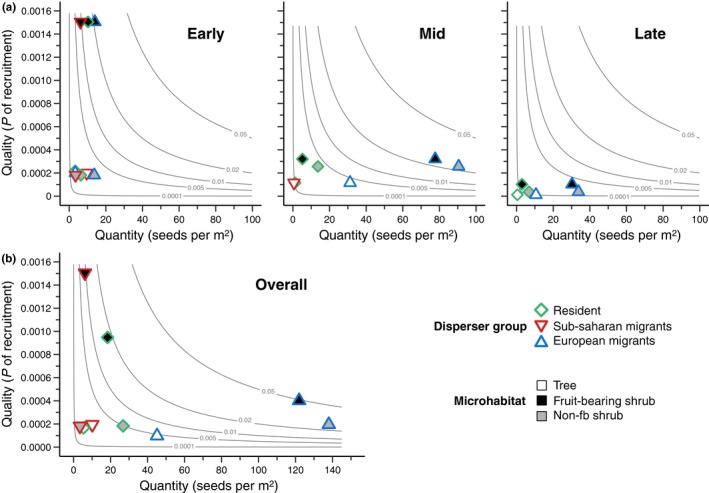
Seed dispersal effectiveness (SDE = Quantity × Quality) landscapes of *Pistacia lentiscus*’ seed dispersers grouped by their migratory strategy (residents, and sub‐Saharan and European migrants) and considering the three studied microhabitats (see legend). Isoclines represent all combinations of quantity and quality components with the same SDE. (a) SDE landscapes per period. (b) Overall SDE landscape [Colour figure can be viewed at http://wileyonlinelibrary.com]

Seed dispersal effectiveness provided by different bird groups (residents, and sub‐Saharan and European migrants) changed remarkably in time due to temporal changes in the quantity and quality components (Figure 4a). The early period was a “low quantity – high quality” period and included the highest quality values obtained across periods and microhabitats (Figure 4a). The disproportionate high quality found beneath fruit‐bearing shrubs resulted from the highest seed viability in the early period along with the highest germination and high seedling survival recorded beneath fruit‐bearing shrubs (Figure 3). The three bird groups contributed to seed deposition in this “microhabitat–period” combination (sub‐Saharan migrants < residents < European migrants). The mid‐period was a “high quantity – intermediate quality” period and included the highest quantity values obtained across periods and microhabitats, mostly contributed by European migrants (Figure 4a). The intermediate quality found in the mid‐period across microhabitats resulted from intermediate levels of seed viability, low germination rates and high rates of seedling survival beneath shrubs (Figure 3). Finally, the late period was a “low quantity – low quality” period (Figure 4a), the low quality mostly resulting from the minimal levels of seed viability in this period (Figure 3). These quantity–quality differences resulted in similar values of total seed dispersal effectiveness (sum across microhabitats and dispersers) in the periods early (SDE = 0.053) and mid (SDE = 0.057), which were one order of magnitude higher than in the late period (SDE = 0.005).

These period‐specific patterns shaped the overall seed dispersal effectiveness across periods (Figure 4b). The overall effectiveness was highest for European migrants dispersing seeds beneath fruit‐bearing and non‐fruit‐bearing shrubs (SDE = 0.049 and 0.027, respectively) followed by resident birds and sub‐Saharan migrants dispersing seeds beneath fruit‐bearing shrubs (SDE = 0.017 and 0.009, respectively) (Figure 4b). Accounting for temporal variation allowed us to unveil a non‐negligible contribution of resident birds and sub‐Saharan migrants to the overall SDE beneath fruit‐bearing shrubs (23.0% and 12.3%, respectively), despite their reduced contribution to the overall quantity component (12.6% and 4.2%, respectively) (Figure 4b).

## 4 DISCUSSION

The effectiveness of seed dispersal mutualisms has been widely explored in previous studies aiming to assess not only the immediate, but also the delayed effects frugivorous animals have on the plant populations they disperse (Schupp, 1993; Schupp et al., 2010). These studies have usually focused on the spatial patterns of seed deposition and seedling recruitment (Calviño‐Cancela & Martín‐Herrero, 2009; Escribano‐Ávila et al., 2014; Jordano & Schupp, 2000; McConkey et al., 2014; Rother, Pizo, & Jordano, 2016), whereas the temporal patterns of seed dispersal effectiveness during the fruiting phenophase have remained completely overlooked. Here, we fill this knowledge gap by demonstrating shifts in the identity and contribution of seed dispersers, the magnitude of seed rain (quantity component) and multiple demographic processes (quality sub components) necessary for seedling recruitment (quality component). We show how small contributions to seed rain by migratory species can result in a relevant effectiveness if they disperse seeds during high‐quality periods for recruitment. These types of temporal shifts in effectiveness are to be expected in dynamic and seasonal environments (Carnicer, Jordano, & Melian, 2009), where plant–frugivore interactions are pivoting around frugivore assemblages with a marked component of migratory and transient species.

### 4.1 The timing of the quantity component

The lentisc is a keystone species of Mediterranean woodlands, occupying central positions in plant–frugivore interaction networks (Olesen et al., 2011), and with a key role as a food item for both generalized frugivores (Jordano, 1988) and insectivores (Jordano, 1987). The contributions of different bird species to the lentisc seed rain were marked by the extreme seasonality and temporal dynamics of the local avifauna. The disperser assemblage actually includes bird species with three distinct migratory strategies (Moreau, 1952; Wernham et al., 2002) that overlay temporally along the lentisc fruiting phenophase, namely residents, sub‐Saharan long‐distance migrants and overwintering species from Northern Europe. Most sub‐Saharan migrants are transient in the study area between summer and early autumn: Only one of the seven species actually breeds in the study site (*Luscinia megarhynchos*; Supporting Information Table S1), and the other six species only use the lowland forests and woodlands of South Spain as stopover sites for fuelling during their autumn migration (Herrera, 1984). This transience explains why the seed rain contribution of sub‐Saharan migrants, mostly contributed by *Ficedula hypoleuca*, was confined to the early period (Figure 2b). In contrast, both the resident species (*Sylvia melanocephala*) and the European migrants (*Erithacus rubecula*,* Sylvia atricapilla* and *Turdus philomelos*) occur in the study area during all or most of the lentisc fruiting phenophase (Supporting Information Table S1). The dispersal peak observed in the mid‐period coincides not only with the ripening peak of the lentisc (Jordano, 1989), but also the massive arrival to the study region of the hyper abundant European migrants, especially *S*. *atricapilla* and *E*. *rubecula* (González‐Varo, 2010; Tellería, Ramírez, & Pérez‐Tris, 2008). European migrants generally stay in their Mediterranean wintering grounds from October to March (e.g., González‐Varo, 2010), which explains the similar seed rain contributions observed in the periods mid (November–January) and late (February–April), despite the lower seed rain densities in the latter.

### 4.2 The timing of the quality component

We analysed four quality sub components of effectiveness and two of them, namely seed viability and germination, varied markedly between periods. The parallel decrease in viability of seeds from fruits and dispersed seeds indicates that such decrease was not caused by the birds, but took place at a pre dispersal stage (Supporting Information Figure S3). As pointed, fruits can remain red in colour because colour is also associated with seed viability and most red fruits contain unviable seeds. This explains why lentisc plants typically bear more red than black fruits at the end of the fruiting season (Jordano, 1989). However, our results show that black fruits are only a reliable signal of seed viability at early and mid‐periods of the fruiting phenophase, because we only tested viability in black fruits and most seeds from the late period were unviable. This suggests that some fruits with unviable seeds might ripen very slowly up to, eventually, acquiring the black colour at the end of the season. As the fruit supply is depleted by the frugivores, the incidence of empty seeds becomes predominant, and such incidence was higher in bird‐dispersed seeds than in seeds sampled from black fruits (Supporting Information Figure S3). The latter suggests that birds could also feed on red fruits despite their preference for the black ones (Jordano, 1989). Unviable seeds include abortion, parthenocarpy or parasitism by the chalcidoid wasp *M. pistaciae* (Jordano, 1989; see also Traveset, 1993) and, thus, the proximate causes underlying viability loss are biotic. We also expected to find temporal differences in post dispersal seed survival, a demographic process governed by the local abundance and foraging preferences of seed predators (García et al., 2007; Ostfeld, Manson, & Canham, 1997), that is, by biotic factors. Unfortunately, our sample sizes did not allow us to detect variation in post dispersal seed predation neither between periods nor between microhabitats. This was unforeseen because similar sample sizes successfully characterized differences in seed predation among populations and between microhabitats in other Mediterranean shrub species (González‐Varo et al., 2012).

Seed germination was the other quality sub component that varied between periods and that variation was higher than that observed between microhabitats. Germination was both lower and slower in the mid‐period, that is, in the seed dispersal peak. Apparently, the main factors underlying temporal differences in germination were abiotic, and there are reasons to think that temperature played a crucial role. The speed and success of germination in lentisc seeds are positively associated with soil humidity (Verdú & García‐Fayos, 1996), but this does not explain the lower and slower germination rates observed in the mid‐period because soil humidity during the first month after sowing was very similar between periods (see details in Supporting Information Figure S5). In contrast, seeds sowed in the mid‐period faced lower temperatures than those sowed in the periods early and late (average air temperature can be 5–8°C lower; see Supporting Information Figure S2). We also expected to find temporal differences in seedling survival between periods because this process can be a demographic bottleneck in strongly seasonal Mediterranean ecosystems due to summer drought (Gómez‐Aparicio, 2008). Yet, we think our limited sample sizes also prevented clearer patterns; we sowed more than 639 seeds, but survival was only assessed in 138 seedlings across nine “microhabitat–period” combinations. Interestingly, the greatest effect in the GLMM analysing seedling survival was accounted by the interaction term, slightly supporting the idea that the quality of microhabitats can vary between periods (see Figure 3d).

### 4.3 The timing of seed dispersal effectiveness

The overlay of the temporally dynamic frugivore assemblage, and the temporally variable seed viability and germination caused heterogeneous effectiveness of lentisc seed dispersers. Intensity of seed rain (maximum in the mid‐period) is temporally decoupled from good conditions for germination (early and late in the season) and seed viability (maximum in the early season). The overall seed dispersal effectiveness was dominated by hyper abundant European migrants, not only owing to their huge quantity contribution throughout the lentisc fruiting phenophase, but also because their quantity contribution was higher than that of residents and sub‐Saharan migrants in the “microhabitat–period” combination with the highest quality for seedling recruitment (i.e., *fruit‐bearing shrubs* – *early*; Figure 4). However, most seed dispersal by sub‐Saharan migrants was uniquely concentrated in that “microhabitat–period” combination, making their mutualistic services to be the ones with the highest overall quality. This evidences that the timing of dispersal can also compensate for quantity inequalities in seasonally dynamic disperser assemblages within seasonal ecosystems. In particular, we would overlook the relevance of sub‐Saharan migrants if we simply consider the overall frugivore contribution to seed rain over the whole fruiting season, or if we assess the quality sub components in the peak or at the end of the fruiting phenophase (e.g., Escribano‐Ávila et al., 2014; García, 2001; González‐Varo et al., 2012).

Interestingly, the effects of timing on the quantity and quality components of effectiveness are expected to vary at wider spatiotemporal scales, between years and among populations. Simply considering the disperser assemblage studied, one would expect that fruit crops should be depleted earlier in years of low fruit production or advanced fruiting phenology. In fact, the lentisc ripening peaks can differ in nearly 1 month between consecutive years (Jordano, 1989), and the local abundance of lentisc fruits in the study site was nearly 10 times greater in the study season than in the previous season (i.e., 2013–2014: J.P. González‐Varo unpublished data). Similarly, fruit crops should be depleted earlier in less dense lentisc populations (see González‐Varo, 2010). Under such scenarios, the relative contributions of Sub‐Saharan migrants could be greater than the ones reported here.

## 5 CONCLUDING REMARKS

Plant–animal mutualisms are intrinsically dynamic forms of ecological interactions. We show here that the timing of plant–frugivore interactions matters for the quantity and quality components of seed dispersal effectiveness. Plants offer a resource provisioning for mutualistic animals, and the seasonal dynamics of animal assemblages along the flowering or fruiting phenophases typically result in a high temporal turnover of interactions (Morente‐López, Lara‐Romero, Ornosa, & Iriondo, 2018). Timing effects on effectiveness are therefore expected to happen in other types of mutualisms like, for instance, pollination. In fact, during the flowering period of a plant, flowers can be exposed to different pollinator faunas differing in the quantity of pollen they can transport (Ivey et al., 2003; Valverde et al., 2016), while pollen germinability (a quality sub component) may depend on local climatic conditions (Aronne, Buonanno, & De Micco, 2015). We think the “quantity–quality” compensatory effects uncovered here for transient sub‐Saharan migrants are likely to occur in highly seasonal environments, where biotic and abiotic conditions change considerably during the whole flowering or fruiting phenophases. We hope our study will foster future research on timing effects on effectiveness of mutualistic interactions and their relevance for ecological functionality and community dynamics.

## AUTHOR CONTRIBUTIONS

J.P.G.‐V. conceived the study, planned the sampling design and collected the data in the field. J.M.A. performed laboratory work. J.P.G.‐V. conducted the statistical analyses and wrote the first manuscript draft. P.J. discussed the idea, the sampling design and the results, and contributed during manuscript writing. All authors approved the final manuscript.

## Data Availability

Data associated with this article (a: seed rain density; b: sample‐level information with seed dispersers identified through DNA barcoding; c: seed viability in ripe fruits; d: seed predation experiment; e: sowing experiment; and f: seed dispersal effectiveness) are deposited in Dryad (https://doi.org/10.5061/dryad.3c4n8nc).
