# Supplementary material for: The timing of frugivore‐mediated seed dispersal effectiveness
Source: Mol Ecol. 2018 Sep 17;28(2):219–31. doi: 10.1111/mec.14850 (PMC6905405; doi:10.1111/mec.14850)
Supplement: Supplementary file 1 [file MEC-28-219-s001.pdf]

Supplemental Information for:

The timing of frugivore-mediated seed dispersal effectiveness

Juan P. González-Varo[1,2] | Juan M. Arroyo[1] | Pedro Jordano[1]

[1]Integrative Ecology Group, Estación Biológica de Doñana, EBD-CSIC, Avda. Américo Vespucio 26, Isla de La Cartuja, E-41092, Sevilla, Spain

[2]Terrestrial Ecology Group, Instituto Mediterráneo de Estudios Avanzados, UIB-CSIC, E-07190, Esporles, Spain

Correspondence: Juan P. González-Varo,

E-mail: jpgvaro@outlook.com

Figure S1. Photographs illustrating different methodological components of the study

Figure S2. Climograph showing monthly mean values (1981–2010) of precipitation and temperature from the nearest climatic station to our study site.

Figure S3. Comparisons across periods between the viability of bird-dispersed seeds and the viability of seeds sampled from black (ripe) fruits.

Figure S4. Frequency distribution of time until germination (weeks after sowing) of seeds sowed in the three study periods (early, mid and late) of the fruiting phenology of *Pistacia lentiscus* (see Figure S2).

Figure S5. Temporal dynamics of soil humidity in the three target microhabitats between October 2014 and May 2015.

Table S1. Number of birds recorded within a fixed-transect in the study forest in monthly birds censuses conducted during the study period.

Table S2. Initial number of sowing stations per 'microhabitat–period' combination and final numbers including data for germination and seedling survival.

Figure S1. Photographs illustrating different methodological components of the study. (a) A lentisc (*Pistacia lentiscus*) shrub. (b–c) Branches of female lentiscs bearing red and black fruits. (d) Lentisc seed. (e) Lentisc seed parasitized by a larva of the chalcidoid wasp *Megastigmus pistaciae*. (f) Empty lentisc seed due to abortion or parthenocarpy. (g) Physiognomy of the study Mediterranean forest, showing a large holm oak tree, tall shrubs and low scrubs. (h) Seed trap placed beneath a kermes oak, a non-fruit-bearing shrub. (i) Lentisc seed defecated by a bird inside a seed trap. (j) One of the seed depots used in the seed predation experiment. (k) Seed depot completely destroyed by rodents. (l) Wire mesh cage used to protect the sowing stations from seed predators; a thin mesh on the top of the cage also prevented the deposition of lentisc seeds into the sowing stations. (m) Sowing stations placed beneath a tree corresponding to the sowings conducted in the periods *early*, *mid* and *late*. (n) Germination of two lentisc seeds. (o) Sowing station with several lentisc seedlings. (p) Sowing station at the end of the experiment, with a surviving seedling on the left and two dead ones on the right.

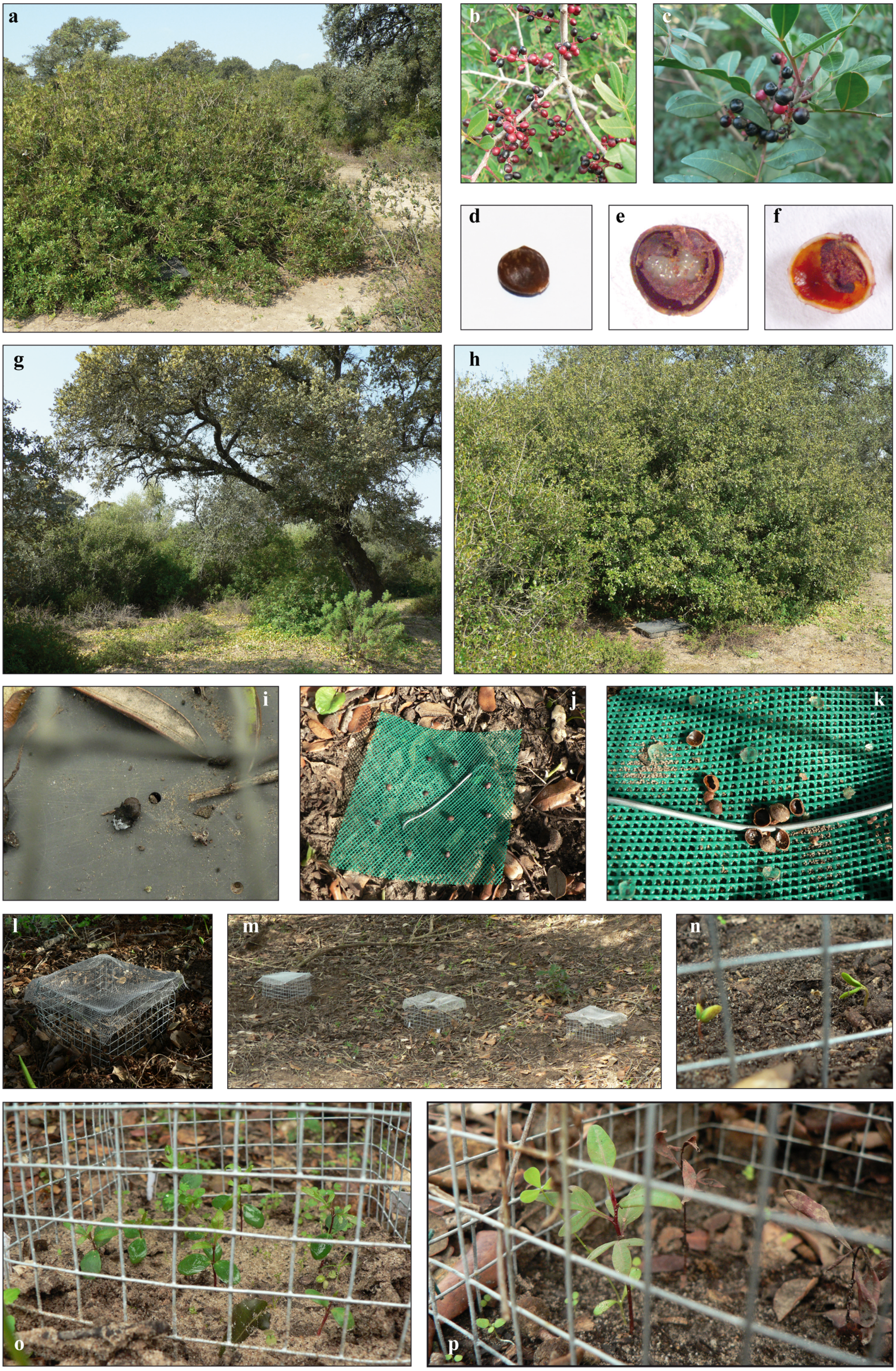
a
b
c
d
e
f
g
h
i
j
k
l
m
n
o
p

Figure S2. Climograph showing monthly mean values (1981–2010) of precipitation and temperature from the nearest climatic station to our study site (~14 km, Jerez de la Frontera airport: 36° 45.0' N, 6° 3.3' W; www.aemet.es). Note that we have ordered the months according to the fruiting phenology of *Pistacia lentiscus*. The upper part of the panel shows the 3-month periods (early, mid and late) by which we divided the whole fruiting phenology. In each period, arrows denote the moments when we collected black ripe (black) fruits and started the field experiments to evaluate seed predation (seed depots), germination and seedling survival (sowings).

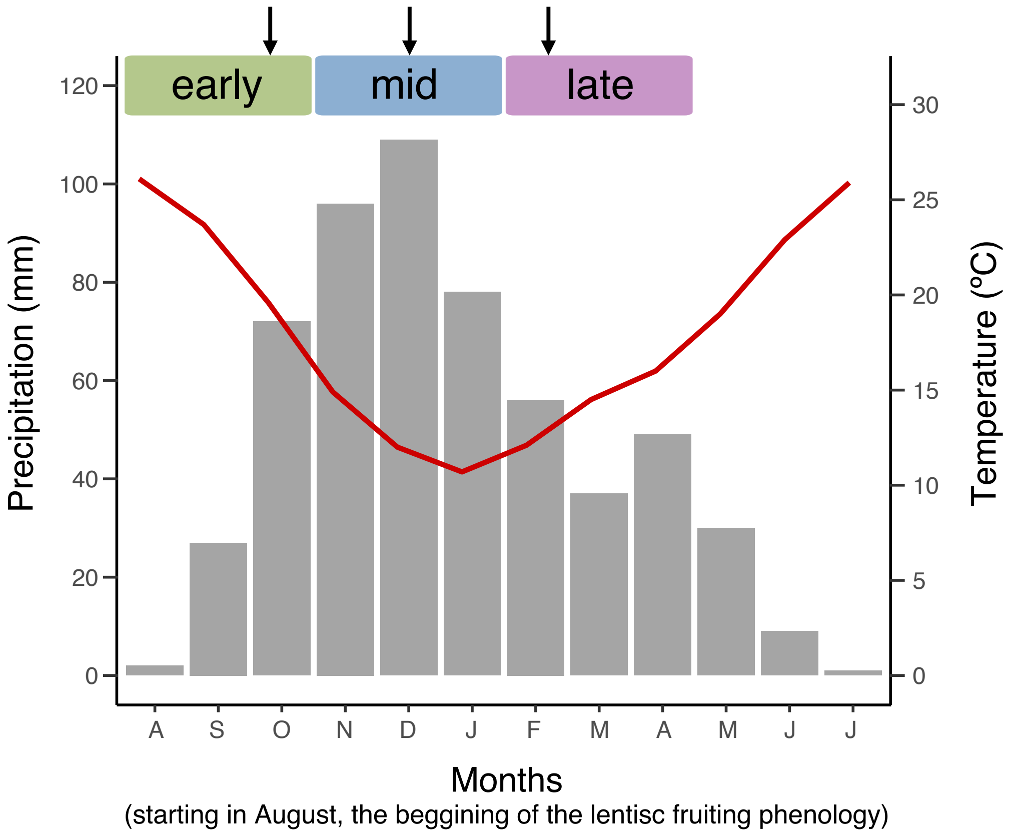

Figure S3. Comparisons across periods between the viability of bird-dispersed seeds (sampled in the seed traps; n = 339, range = 60–182 per period) and the viability of seeds sampled from black (ripe) fruits (n = 2288, range = 555–925 per period). In both cases, seed viability was tested through the 'flotation/sink' method (see details in section 2.5 of the main text). Note that the viability values of bird-dispersed seeds correspond to those shown in Figure 3a of the main text.

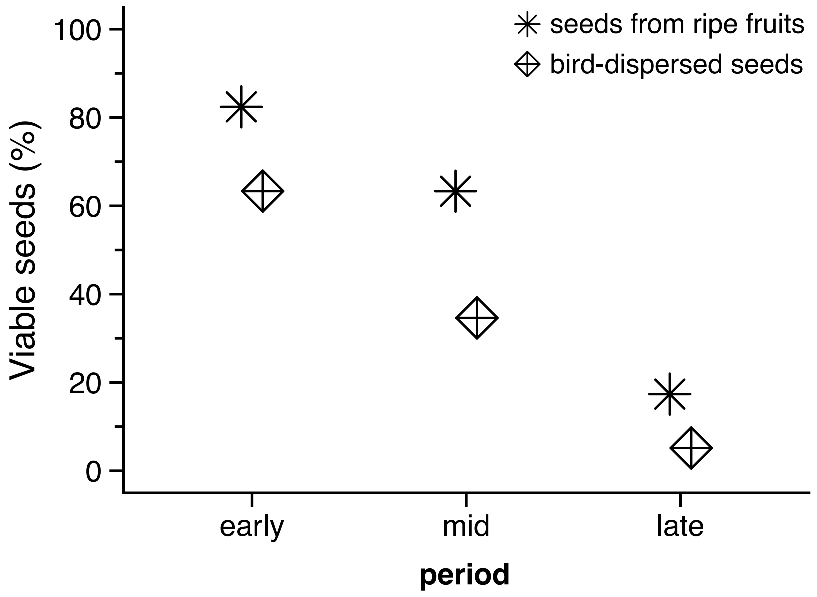

Figure S4. Frequency distribution of time until germination (weeks after sowing) of seeds sowed in the three study periods (early, mid and late) of the fruiting phenology of *Pistacia lentiscus* (see Figure S2). These times differed significantly between periods (Kruskal-Wallis test: $\chi^2 = 48.7$, $P = 2.6 \times 10^{-11}$). Germination times in periods early and late did not differ significantly (Mann-Whitney U test: $P = 0.295$), but both differed significantly from times in the mid period (Mann-Whitney U test: $P < 7.0 \times 10^{-8}$ in both cases). A plausible explanation for this pattern is the lower temperature faced by seeds sowed in the mid period as compared to those sowed in the periods early and late (see Figure S2).

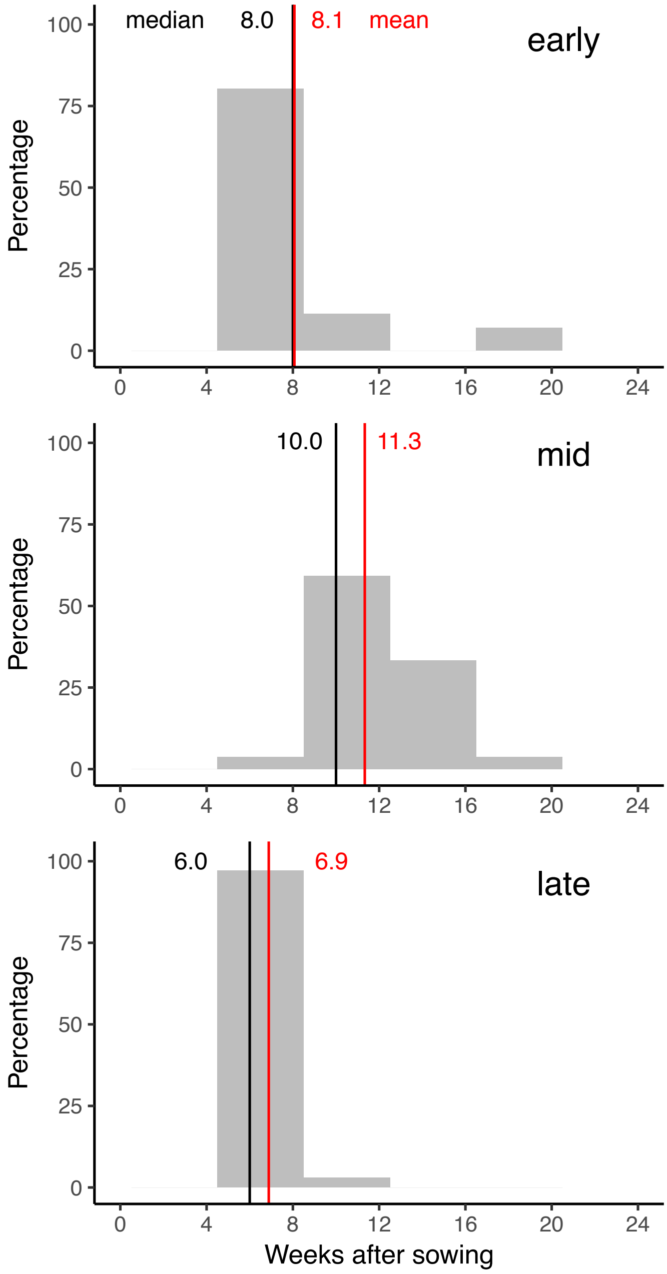

Figure S5. Temporal dynamics of soil humidity (mean ± 95% CI volumetric water content [VWC]) in the three target microhabitats between October 2014 and May 2015 (nine measurements). Arrows with labels denote the date of the sowing experiments for periods early, mid and late. We measured VWC with a time-domain reflectometer (TDR, Campbell Scientific Inc., Logan, UT, USA) with 12-cm depth rods in 8–13 points per microhabitat type). We did not measure VWC through the summer because values were always <5%, which – in the sandy soils of the study site – resulted either in errors or in unreliable estimates. A previous study showed that the minimum VWC values recorded in eight lowland forest patches of this region occurred during the summer (see Appendix E in González-Varo et al., 2012 *Perspectives in Plant Ecology, Evolution and Systematics* 14: 111–122).

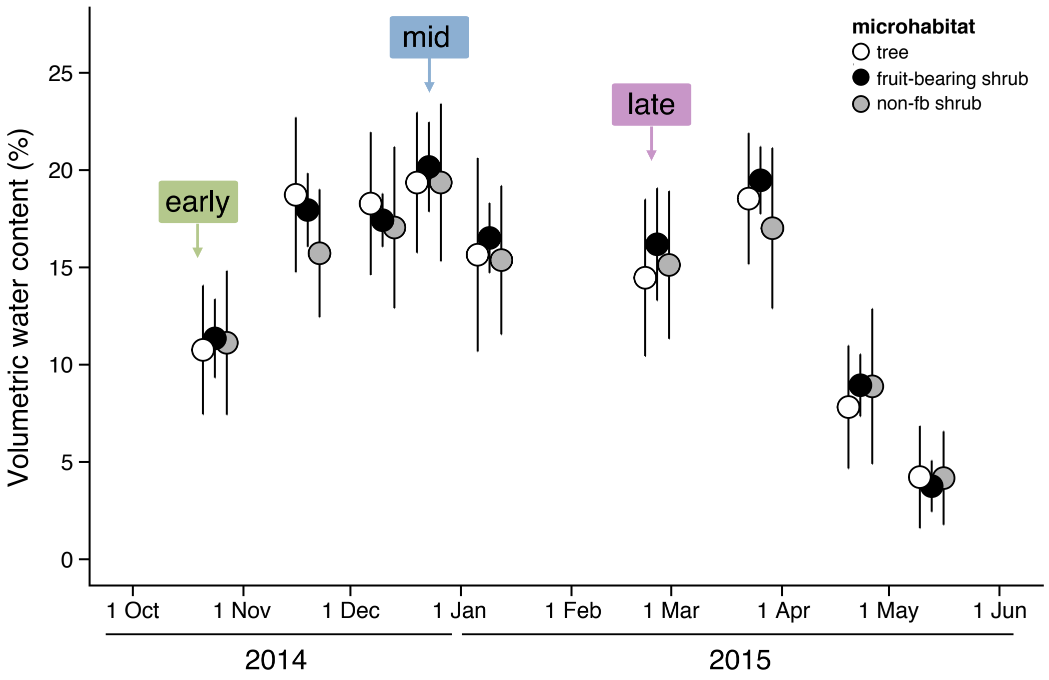

Table S1. Number of birds recorded within a fixed-transect in the study forest (600 m length and 20 m width along each side; 600 m × 40 m = 2.4 ha) in monthly birds censuses conducted from August 2014 to April 2015. (A) Bird species (family) identified in lentisc seeds through DNA barcoding and classified according to their migratory strategy (see details in the main text); months with counts are shaded to illustrate phenological differences. Note that two sub-Saharan migrant species (*) identified through DNA barcoding were not detected by bird censuses. (B) Other bird species (family) recorded during the study, sorted alphabetically; air foraging species from Hirundinidae (swallows and martins), Apodidae (swifts) and Meropidae (bee-eaters) were not considered in the censuses.

(A) Species identified through DNA barcoding	Aug	Sep	Oct	Nov	Dec	Jan	Feb	Mar	Apr
Resident									
Sylvia melanocephala (Sylviidae)	33	30	22	22	26	24	21	23	27
Sub-Saharan migrants									
Luscinia megarhynchos (Muscicapidae)	13	5							50
Ficedula hypoleuca (Muscicapidae)		27	3						
Muscicapa striata (Muscicapidae)		2	31						
Phoenicurus phoenicurus (Muscicapidae)		2							
Sylvia borin (Sylviidae)	2	16							
*Sylvia communis** (Sylviidae)									
*Sylvia hortensis** (Sylviidae)									
Intra-European migrants									
Erithacus rubecula (Muscicapidae)			34	27	30	15	6	2	
Sylvia atricapilla (Sylviidae)			21	41	49	55	23	7	
Turdus philomelos (Turdidae)				8	17	14	7	5	

(B) Bird species recorded during the study	Aug	Sep	Oct	Nov	Dec	Jan	Feb	Mar	Apr
Accipiter nisus (Accipitridae)				1					
Alectoris rufa (Phasianidae)				6				3	
Buteo buteo (Accipitridae)									
Carduelis carduelis (Fringillidae)	8	14					6		1
Carduelis chloris (Fringillidae)	17	10	8			5	3	12	1
Certhia brachydactyla (Certhiidae)	5	1						2	
Columba palumbus (Columbidae)			4			1	3	5	2
Corvus monedula (Corvidae)		3	2				4	6	13
Cyanistes caeruleus (Paridae)	11	5	2	3	4	2	2	2	5
Elanus caeruleus (Accipitridae)				1					
Falco tinnunculus (Falconidae)			1				1		
Fringilla coelebs (Fringillidae)	2		2	3	3	1	5		
Oriolus oriolus (Oriolidae)	1								
Parus major (Paridae)	1	2	2		2	4	1	2	1
Phoenicurus ochruros (Muscicapidae)						1			
Phylloscopus collybita (Phylloscopidae)		2		3	9	7	3	1	
Picus viridis (Picidae)	3	2	4	1	2	2	2	1	1
Serinus serinus (Fringillidae)	1	2	3	1		1	24	8	8
Turdus merula (Turdidae)	2	1		1	2	1	1	1	
Upupa epops (Upupidae)	1								

Table S2. Initial number of sowing stations per 'microhabitat–period' combination and final numbers including data for germination and seedling survival. The lost of sowing stations was due to damage by wild boars (*Sus scrofa*), which were particularly active in our sampling area during the early period. Differences between germination and survival data depended on when the damages took place. For example, if a sowing station was damaged only a few weeks after sowing (e.g. after 6 weeks), it was excluded from both the germination and the survival data. However, if a sowing station was damaged many weeks after sowing (e.g. after 16 weeks, when most seeds had germinated; see Figure S4), we kept it in the germination data but excluded it from the survival data. Germination data was lost in 9 damaged/removed sowing stations (range = 0–2 per 'microhabitat–period' combination) and survival data in 18 (range = 0–6).

Period	Microhabitat	Initial *n* sowing stations	Germination data	Seedling survival data
early	tree	10	8	4
	fruit-bearing shrub	8	7	6
	non-fb shrub	8	6	5
mid	tree	8	6	5
	fruit-bearing shrub	7	6	6
	non-fb shrub	7	6	6
late	tree	9	9	9
	fruit-bearing shrub	7	7	6
	non-fb shrub	7	7	6
TOTAL		71	62	53
